# Nanolithography using Bessel Beams of Extreme Ultraviolet Wavelength

**DOI:** 10.1038/srep31301

**Published:** 2016-08-09

**Authors:** Daniel Fan, Li Wang, Yasin Ekinci

**Affiliations:** 1 Paul Scherrer Institute, 5232 Villigen-PSI, Switzerland

## Abstract

Bessel beams are nondiffracting light beams with large depth-of-focus and self-healing properties, making them suitable as a serial beam writing tool over surfaces with arbitrary topography. This property breaks the inherent resolution vs. depth-of-focus tradeoff of photolithography. One approach for their formation is to use circularly symmetric diffraction gratings. Such a ring grating was designed and fabricated for the extreme ultraviolet (EUV) wavelength of 13.5 nm, a candidate wavelength for future industrial lithography. Exposure of the aerial images showed that a Bessel beam with an approximately 1 mm long z-invariant central core of 223 nm diameter had been achieved, in good agreement with theory. Arbitrary patterns were written using the Bessel spot, demonstrating possible future application of Bessel beams for serial beam writing. Lithographic marks of ~30 nm size were also observed using a high resolution Bessel beam.

Nondiffracting beams have attracted substantial attention since their discovery in the 1980s^1^. Nondiffracting, diffraction-free, or *z*-independent solutions of the Helmholtz equation are indeed possible for various wave forms, such as Bessel beams, Mathieu beams, and Weber beams^2^,^3^. They are defined by the fact that their transverse intensity profile is invariant under free-space propagation. Nondiffracting beams have unique properties such as large depth-of-focus and self-healing of the central core^4^, whereby the central core re-constructs or re-forms after being obstructed, making them attractive for applications such as optical trapping^2^,^5^, electron accelerators^6^, bio-imaging^7^, laser ablation^8^, lithography^9^,^10^, and materials processing^11^.

The Bessel beam is the simplest form of a nondiffracting beam, where its amplitude is proportional to the zeroth-order Bessel function of the first kind, which is a sharp peak surrounded by rings, not changing along the propagation axis^4^. Approximations to nondiffracting Bessel beams have been experimentally observed by focusing a Gaussian wave with an axicon lens^12^, using annular slits in the far field^1^, holographic methods^13^, by use of metamaterials and nanowire media^14^,^15^, and by circularly symmetric diffraction gratings^16^. Depending on the synthesis method, the beam can be chromatic or achromatic^12^. It has been shown that Bessel beams can be developed as a limiting case in the family of discrete nondiffracting beams^17^,^18^,^19^,^20^.

Some of the interesting and useful properties of Bessel beams are their ability to self-heal and their propagation invariant central core. This means that the central core is diffraction resistant and has a steady intensity along the length of the beam, making it suitable for serial beam lithography, as it allows exposure over topographies of varying heights while keeping the beam spot size and intensity constant. Therefore, for serial beam writing, it is desirable to have a long central beam core and increased working distance.

In general, interference of multiple beams with the same transverse wave vector and with sufficiently large beam size generates periodic and aperiodic aerial images which are nondiffracting. Consider two plane-wave beams with wavelength *λ* converging at a propagation angle of *θ* along the *z*-axis as shown in Fig. 1. The beams are placed circularly symmetric to the *z*-axis at a position *r* from the origin. In this case the beam will interfere when they overlap and form an aerial image. The working distance, thereafter referred to as the ‘gap’ (*g*), between the source plane and image plane is given as


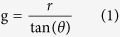


The total intensity of the image produced by the interference of the coherent beams of the same intensity *I*_0_, is


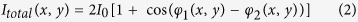


For two sources positioned along the *x*-axis, with the transverse *k*-vector 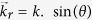, the aerial image is given as


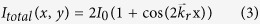


This represents a sinusoidal line/space pattern with a period of 2*π*/2*k*_*r*_. It is clear that the intensity distribution has no *z*-dependence, and thus is nondiffracting.

One can increase the number of interfering beams at a transverse distance ‘*r*’ from the normal axis. For example, by placing four beams 90 degrees to each other in a diamond formation, an interference pattern consisting of holes or dots can be formed^21^. Using 6 beams and adjusting the phase difference, periodic patterns such as honeycomb and kagome lattices can be obtained^22^. Using 5 or 8 beams, quasi-periodic patterns are formed^23^. As the number of beams approaches infinity in a ring arrangement, the aerial image becomes a zeroth-order 2D Bessel function^18^ given as


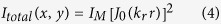


where 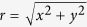 and *I*_*M*_ is the maximum intensity of the diffracted light. Again, the aerial image is *z*-invariant where the interfering beams overlap. This overlapping region has a certain depth that can be estimated as (considering Fig. 1)


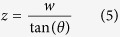


where *w* is the width of the interfering beams, i.e. the width of the annular ring, which forms the effective source. Within the range of *z*, the intensity is *z*-invariant. As can be seen, the Bessel beam is a special case of *z*-independent aerial images formed by the interference of multiple beams that have the same *k*_*r*_ value. One can create various forms of nondiffracting beams by changing the number of beams and their relative phases^18^.

A simple method of forming multiple beams is by using diffraction gratings. The diffraction angle (*θ*) of the grating and the grating period (*P*) are related by the Bragg equation:


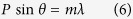


where *m* is the diffraction order and *λ* is the incident wavelength of light. Alternatively, the radial *k*-vector can be written as


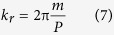


It should be noted that the radial wave vector intensity which defines the aerial image for multiple beam interference, analytically given for line patterns and Bessel beam in equations 3 and 4, becomes independent of wavelength and depends only on the period of the diffraction grating and diffraction order. Therefore the use of annular ring gratings enables achromatic Bessel beams. The radius of the central core is given as^2^:


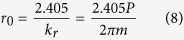


For example, given a ring grating with period 40 nm, a Bessel spot of approximately 30 nm full-width at half maximum (FWHM) will be produced. The theoretical limit occurs when the diffraction angle becomes 90°, whereby following equations 6 and 8 the Bessel spot would have a FWHM of ~10 nm for the 13.5 nm incident wavelength. The depth-of-focus according to equation 5 is dependent on the width of the ring and therefore is practically limited by the spot size of the coherent source. For example to produce a 15 nm diameter spot using EUV corresponds to a grating with 20 nm pitch and 42° diffraction angle. For a 1 mm depth-of-focus the width of the annular ring needs to be ~932 μm wide, and therefore the EUV spot size needs to be at least 2 mm diameter, which is a reasonable number. Therefore, circular gratings will enable an intense Bessel beam with very narrow waist and much extended depth-of-focus, as given in equation 5, using broadband sources as compared with Gaussian beams^24^. These properties are particularly advantageous for photolithography. In particular, depth-of-focus is a significant problem in photolithography where the resolution is given as *λ*/2sin*θ* and the depth-of-focus is *λ*/sin^2^*θ*. A nondiffracting beam with large depth-of-focus allows writing of arbitrary structures over topographical surfaces and breaks one of the fundamental trade-offs of photolithography.

The resolution of lithography using Bessel beams is defined in equation 8. *P* has to be larger than λ and the resolution is ultimately limited by the wavelength. Therefore to increase the resolution it would be desirable to use shorter wavelengths. The current state-of-the-art photolithography uses the deep-UV (DUV) wavelength 193 nm, and combined with techniques such as multiple patterning and immersion, features below 20 nm have been patterned. A method to improve resolution is to use the shorter, extreme UV (EUV) wavelength of 13.5 nm. Indeed, it is the outstanding candidate considered by industry, due to the availability of efficient optics, sources, and photoresists^25^. EUV interference lithography has been demonstrated to achieve resolution down to 7 nm half-pitch^26^.

By adapting the approach of diffraction-based EUV-IL^27^, we use multiple-beam interference in the limiting case for the generation of a Bessel beam, and demonstrate its use as a serial beam writing tool. As shown in Fig. 2, Bessel beams have a large depth-of-focus without a compromise in resolution, allowing exposure of arbitrary structures on topographic surfaces.

## Methods

**Figure 3:**
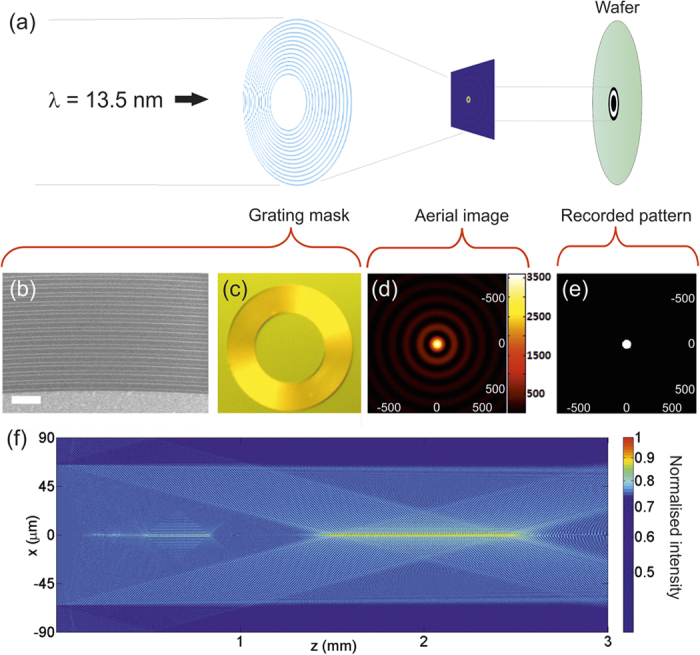
Schematic of Bessel beam formation using ring grating. (**a**) Coherent EUV light is incident from the left and passes through a spatial filter. It is then diffracted by the mask to form a Bessel beam, whose image is recorded in photoresist. (**b**) SEM and (**c**) optical image of the grating mask (scale bar = 1 μm). (**d**) Simulation of the Bessel beam aerial image (with intensity scale in arbitrary units) and (**e**) after application of a threshold, corresponding to exposure in photoresist. Scale in the *x* and *y* axes in nanometers. (**f**) Simulation of the Bessel beam propagation with normalised intensity scale.

A schematic of the experimental arrangement is shown in Fig. 3a, where spatially coherent light at the EUV wavelength of 13.5 nm passes through a spatial filter and then onto a ring grating mask where the light is diffracted and interferes to form a Bessel beam. This also demonstrates how Bessel beams are composed of a set of plane waves propagating on a cone.

Our design has a transmission diffraction ring grating with 300 nm period. The inner diameter is 130 μm and outer diameter 230 μm. This gives a ring width of 100 μm which is less than the inner diameter, thus avoiding zeroth order overlap. The grating period gives a diffraction angle of 2.58° and an optimum gap between mask and sample of 2 mm, as shown in Table 1. The central core radius r_0_ is then calculated to be 115 nm, with a *z*_*max*_ of 2.56 mm as calculated from equations 1, 5, and 8. The mask fabrication process is described below, while scanning electron microscope image and optical image of the mask are shown in Fig. 3b,c respectively. Simulations of beam propagation were carried out using MATLAB software (R2014a, Mathworks Inc., Natick MA, USA), with optical constants from the literature^28^, and the Bessel beam cross-section in the transverse plane is shown in Fig. 3d. No rigorous light-matter interactions are modelled in the ring grating as the periodicity is large enough compared to the incident wavelength to use the scalar diffraction approximation. Simulations of the application of photoresist dosage thresholds were also performed in MATLAB, shown in Fig. 3e. This simulates the recorded image in photoresist for various EUV exposure dosages by applying a hard threshold. Figure 3f shows a simulation of the Bessel beam propagation for the 300 nm period diffraction ring from 0 to 3 mm in the *z*-direction. The intensity was plotted on a logarithmic scale to better show the fine structure of the aerial interference pattern, and in particular the Bessel side rings can be clearly seen. The Bessel beam due to the first order diffraction is clearly seen between 1.45 and 2.56 mm. For a perfectly binary grating, the 2^nd^ order diffraction is zero, and therefore no Bessel beam due to 2^nd^ order diffraction was observed in the simulation.

The designed mask was fabricated on a 100-nm-thick Si_3_N_4_ membrane. Base layers of 5 nm chromium (Cr), 10 nm gold (Au), and 8 nm Cr were thermally evaporated (BAE 250, Oerlikon Balzers, Liechtenstein) on the membrane. The first 5 nm of Cr was used as an adhesion layer between the membrane and the Au layer. The 10 nm of Au was used as a subsequent electroplating seed layer. The last 8 nm of Cr was used as a conduction layer during e-beam writing to avoid charging effects. Hydrogen silsesquioxane (HSQ, Dow Chemical Company, Midland, MI, USA) FOX 16 photoresist diluted at 1:1 with methyl isobutyl ketone (MIBK) was spin-coated at 2500 rpm for 60 s to give a target thickness of 250 nm. The circular grating pattern was written by an e-beam lithography tool^29^ (EBPG 5000+, Vistec, Jena, Germany) at a dose of 8000 μC/cm^2^. Subsequently, the sample was developed in a NaOH buffered solution (MICROPOSIT™ 351, Rohm and Haas, Philadelphia, PA, USA), diluted 1:3 with deionized water for 120 s and then rinsed with deionized water and dried with nitrogen blow. The 8 nm of Cr was etched away in a Cl_2_ plasma etcher (BMP Plasmatechnologie, Garching, Germany) for 30 s to reveal the Au electroplating seed layer. The mask was then electroplated with 150 nm of Au to form the grating lines.

A second, high-resolution Bessel mask was also fabricated with a ring diameter of 300 μm, ring width of 100 μm, and ring grating pitch of 40 nm. Using equations 1, 6 and 8 gives a diffraction angle of 19.7°, a mask to sample gap of 418 μm, a depth-of-focus of 279 μm, and a spot size of ~30 nm. The mask was fabricated by spin-coating HSQ (XR1541) at 3000 rpm for 45 s on a 100 nm thick SiN membrane followed by e-beam lithography. The developed HSQ gratings are the final grating material, and no electroplating was involved as such high resolution features are difficult to electroplate. Development was done using Microposit 351 diluted 1:3 with DI water for 30 s. A second e-beam overlay was performed to fabricate the central photon stop. Poly methyl methacrylate (PMMA) with molecular weight 950k dissolved in 4% ethyl-lactate was spin-coated onto the membrane at 2000 rpm for 45 s targeting a thickness of 400 nm. The areas around the grating ring were exposed by e-beam lithography and subsequently removed by development in iso-propanol mixed 7:3 with DI water for 20 s. Cr (5 nm) and Au (10 nm) as electroplating seed layer were then deposited using thermal evaporation and the mask placed in acetone to lift-off the PMMA on the ring grating areas, leaving the thin metals in areas without the ring grating. Nickel electroplating was performed in a nickel sulfamate/boric acid based bath (Lectro-nic 10-03, Ethone, West Haven CT, USA) using monophasic pulses of ~2.5 mA/cm^2^ current density, 67% duty cycle, 2 s cycle time and plating time of 7 min to grow a 400-nm-thick nickel layer in areas without the ring grating, to act as a photon stop.

The EUV exposures were performed at the XIL-II beamline of the Swiss Light Source (SLS) at the Paul Scherrer Institut (Villigen, Switzerland) using EUV light of 13.5 nm wavelength with 4% bandwidth. The EUV-IL exposures were recorded on HSQ resist XR1541, spin-coated at 2500 rpm (5000 rpm for the high resolution exposures), and developed by the NaOH buffered solution (Microposit 351) diluted 1:3 with deionized water for 30 s.

## Results and Discussion

**Figure 4:**
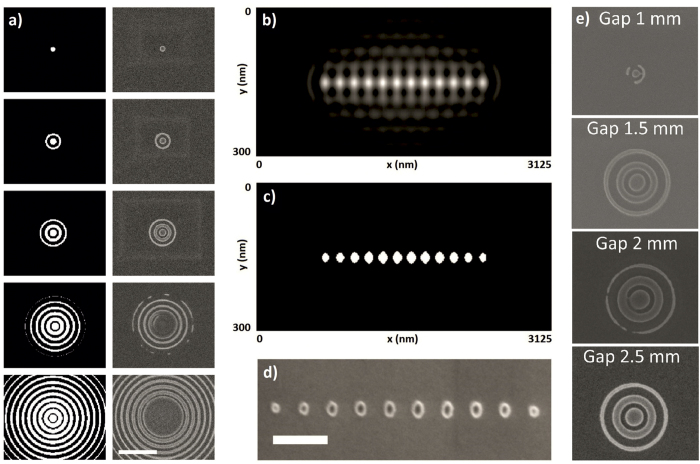
(**a**) Simulation (left) and experimental (right) of Bessel beam at various thresholds/doses. The experimental doses (dose before the grating) in HSQ from top to bottom are 12.1, 23.1, 39, 74, and 124.9 mJ/cm^2^, and match well with simulation. Scale bar is 1 μm. Fluctuations in the simulation are due to simulation mesh size. (**b**) Simulation of Bessel beam intensity showing artefacts and proximity effect due to side rings. (**c**) Applying a threshold by changing the resist dosage can mitigate the problem. (**d**) Experimental exposure in HSQ, scale bar = 300 nm. The dark central region in the spots is due to difference in electron scattering between flat areas and edges under SEM, and is an artefact of the SEM micrograph. (**e**) Exposed pattern at various gap distances.

Figure 4 shows the developed images of the recorded Bessel beam using a scanning electron microscope (Supra V55, Zeiss, Oberkochem, Germany). In Fig. 4a, exposures with different doses on the right and the simulated image on the left are shown. The recorded intensity as the dose is increased agrees well with the simulations. For smaller doses, only the central beam is recorded. With increasing dose, more and more of the side rings become exposed. This corresponds well with simulation of the Bessel beam sectional profile (Fig. 4a, left). The top exposure in Fig. 4a corresponds to a dose of 12 mJ/cm^2^ (i.e. dose on mask), while the first side ring appears at 23 mJ/cm^2^. The FWHM of the central beam at high dose was measured to be approximately 223 nm diameter, in comparison with the theoretical 230 nm.

Although we were, by using the correct dose, able to obtain a single Bessel spot, it may be possible that a line of such Bessel spots would exhibit a proximity effect at the central spots (Fig. 4b–d) due to dosage contribution from neighbouring Bessel beams. Figure 4b shows a simulation of the aerial image of a row of Bessel beams. Note that the side ring intensities in the aerial image are quite apparent. To mitigate these side rings, we can use a high contrast photoresist which captures the central beam spot but is insensitive to the lower intensity side rings. By applying a threshold which simulates resist sensitivity (Fig. 4c) we can determine the Bessel beam exposure dosage which gives only a central spot and where the side rings are not recorded. HSQ is a high contrast, inorganic, and negative-tone photoresist. By patterning in HSQ (Fig. 4d), we observe the exposure of the line of Bessel beam central spots without the surrounding side rings, in accordance with the simulations.

However, note that in Fig. 4c,d, the middle spots are larger than the side spots. This is due to the contribution of the side rings of neighbouring Bessel beams to the dosage in the spot area. For photoresists, any slight exposure to radiation or energy beyond the activation energy will cause chemical changes and thereby lower the subsequent required dosage during lithography. Although the side rings themselves are not recorded, the intensities of their aerial image nevertheless contribute to the exposure dose in the spot areas. When radiation from a Bessel side-ring slightly exposes neighbouring regions, subsequent exposure doses in these areas change leading to overexposure and increase in the feature size. This is analogous to the proximity effect in electron beam lithography, where secondary electrons cause resist exposure in unwanted areas, setting limitations in writing dense patterns. To mitigate this problem careful adjustment of the dosage or proximity correction is needed. For example, in the case of Fig. 4d, the middle spots would need to be under-dosed compared with the spots at either end in order for all the spots to have the same size. The small changes in dose that is required can be easily simulated in software.

The EUV-IL exposures for various gaps are shown in Fig. 4e. The Bessel beam cross-section is clearly shown for gaps 1.5 mm, 2 mm, and 2.5 mm, which corresponds to the beam formed by 1st order interference. The size of the central dot in each case was measured to be 223 nm ± 5 nm. The central spot and two side rings have the same shape and intensity for these three gaps, which shows that the Bessel beam is nondiffracting within the calculated minimum to maximum range as per Table 1. A smaller Bessel beam was also observed at a gap of 1 mm, which corresponds to the location of the optimal distance to observe a beam due to 2nd order interference. Although from the simulation in Fig. 3f, no beam due to 2^nd^ order interference is predicted for a perfectly binary grating, the fabricated grating is not perfectly binary, and therefore some increase in the 2^nd^ order diffraction efficiency is expected. As predicted, the beam size is much smaller with a measured diameter of 114 nm, and in fact the beam spot is one half the FWHM of the beam spot due to first order interference, calculated to be 115 nm as per equation 8. These exposures show that Bessel beam formation using the transmission ring grating approach agrees well with theory.

**Figure 5:**
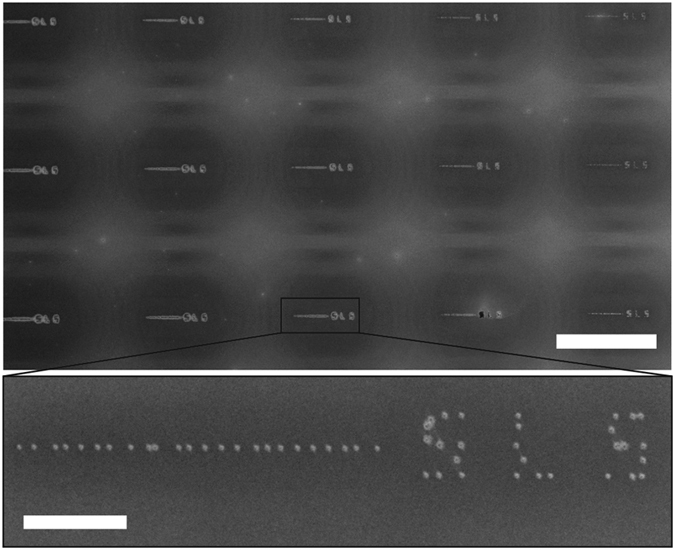
Serial beam writing using Bessel beam array formed from EUV light. The 53 spots were written in 39.75 s using a beam flux of 4 mW/cm^2^. Top scale bar is 100 μm. Inset scale bar is 10 μm.

We also demonstrate the use of the Bessel beam as a serial EUV beam writer. The beam spot shape and intensity is well repeatable, as seen in Fig. 5. The variability comes from inaccuracies in the sample stage, which would be alleviated by using a precise, laser-interferometer controlled stage, which would be able to place lithographic marks more accurately. Our sample stage has a position tolerance of ±500 nm. Mechanical drift and vibrations may also play a role in the variabilities of spot placement. This shows the ability of using Bessel beams to write arbitrary, nano-sized structures, across varying topographies. Since it is a serial writing method the throughput is limited, although large arrays of Bessel beams could be used to increase throughput (Fig. 5). The mask-to-gap distance in this case was set to 1 mm to record the Bessel beam due to 2^nd^ order diffraction, which has a theoretical spot size of 115 nm FWHM. Nineteen measurements of the spot size were taken using SEM giving an average diameter of 124 nm with a standard deviation of 13.4 nm. Further measurement errors are expected due to the SEM measurement method. Continuous lines were not written due to deficiencies in our experimental setup with respect to stage motor accuracy and shutter speeds, although in theory with more accurate control over stage position and shutter times continuous lines can be written.

**Figure 6:**
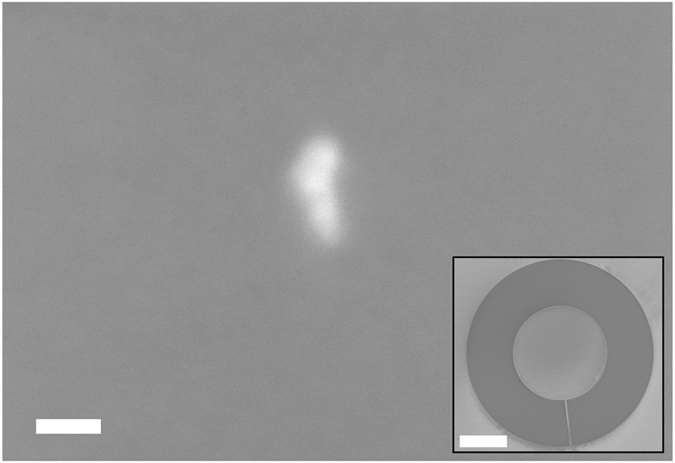
SEM image of a 30 nm × 20 nm lithographic mark in HSQ exposed by interference of diffracted EUV beams from a 40 nm pitch HSQ ring grating. Scale bar is 20 nm. Inset: SEM image of the HSQ ring grating with nickel photon stop on a silicon nitride membrane. Scale bar is 100 μm.

Finally, high resolution dots with 30 nm diameter were exposed, with the results shown in Fig. 6. An exposed dot of 30 nm × 20 nm size was found in the middle of the exposure field as expected. The mask-to-sample gap distance was 550 μm which is within the range of the calculated depth-of-field for 1^st^ order interference Bessel beam. The dose was 268 mJ/cm^2^, which is ten times higher than the dose required for the gold-based ring grating of 300 nm pitch. This is due to losses in diffraction efficiency in the HSQ material at 40 nm pitch and EUV wavelength.

There are a number of reasons which might explain why the dot is astigmatised and not of an ideal shape, and also why such an experiment is currently difficult to perfect. Firstly, at such high resolutions the diffraction efficiencies of the ring grating at different positions of the grating will be different due to the polarization of the incident light. The EUV beam that was used was linearly polarized, meaning that diffraction efficiencies from the ring grating at positions on the vertical axis will be slightly different compared to the horizontal axis. Adjustment of the duty cycle of the ring grating at various positions along the ring will be needed to ensure that linearly polarized light is diffracted with equal intensity at all positions. Secondly, the diffraction angle for 40 nm pitch gratings is 19.7°. At such high diffraction angles combined with the linearly polarized nature of the light, the interference intensities due to diffraction from various parts of the ring will be different. Therefore, compensation for polarization effects will be needed in future ring grating designs.

For high resolution features of 30 nm dot size, the mask to sample gap is much smaller and the exposure is more sensitive to mask inhomogeneities, beam inhomogeneities, mechanical vibrations and drift, and sample stage and mask holder positioning tolerances. Adhesion of the photoresist on the sample surface can also become a problem at such high resolution. Bessel beam diffraction masks are also very challenging to fabricate. For a 30 nm spot the ring grating period required would be 40 nm and any defects or inhomogeneities in the mask would be amplified on the sample, as can be seen in the irregular dot shape in Fig. 6. The technology to fabricate high resolution gratings using e-beam is constantly being improved and 40 nm pitch gratings in HSQ represent the resolution limit for our lab. Lastly, a 30 nm sized dot is quite difficult to find on a sample for SEM inspection. The position tolerance for our interference lithography sample stage is ±500 nm. An improved sample stage such as a piezo-actuated stage or laser-interferometric stage would improve the placement of such lithography marks.

## Conclusions

We have demonstrated the formation of a Bessel beam of EUV light using a transmission ring grating. The formed beam was recorded in photoresist, which has a much higher resolution compared with optical detection systems such as CCD cameras. For various gaps, the recorded pattern intensity corresponds well with theoretical Bessel beam propagation. Bessel beams due to 2nd order interference was also observed. By varying the exposure dose, we can adjust the beam intensity profile that is recorded. This is important for applications in beam writing where the correct dose is needed to avoid artefacts, proximity effect, and unwanted exposure of side-rings. Beam writing was also demonstrated showing good reproducibility, and accuracy could be increased by using a more precise sample stage. Compared to Fresnel zone-plate (FZP) lithography which is a similar scheme, Bessel beam lithography can produce similar spot sizes but with a much larger depth-of-focus^30^. Therefore, Bessel beam lithography can overcome one of the main tradeoffs in traditional photolithography of resolution vs. depth-of-focus, by forming high resolution beams with very large depth-of-focus. Using our current technology as described in this work, a Bessel spot of ~30 nm with a depth-of-focus of ~1 mm would be achievable. Bessel beams show good promise as a serial beam writing tool for arbitrary nanostructures over different surface topographies and curved surfaces, for example as an alternative to nanoimprint lithography^31^ and microlens arrays^32^ for patterning spray-coated curved surfaces. Astigmatism of the Bessel beam due to polarization effects can be mitigated in future designs while astigmatism due to incidence angle between the beam and the recording surface can limit the curvature of the sample that can be patterned as well as the beam deflection. Multichannel arrays of Bessel beams can be used to increase throughput^33^, while the Bessel beam side-lobes could be further suppressed by using another hollow Bessel beam^34^,^35^. In summary, Bessel beam lithography is an effective method of nanolithography for applications in which conventional methods fail due to limited depth of focus.

## Additional Information

**How to cite this article**: Fan, D. *et al.* Nanolithography using Bessel Beams of Extreme Ultraviolet Wavelength. *Sci. Rep.* **6**, 31301; doi: 10.1038/srep31301 (2016).

## Figures and Tables

**Figure 1:**
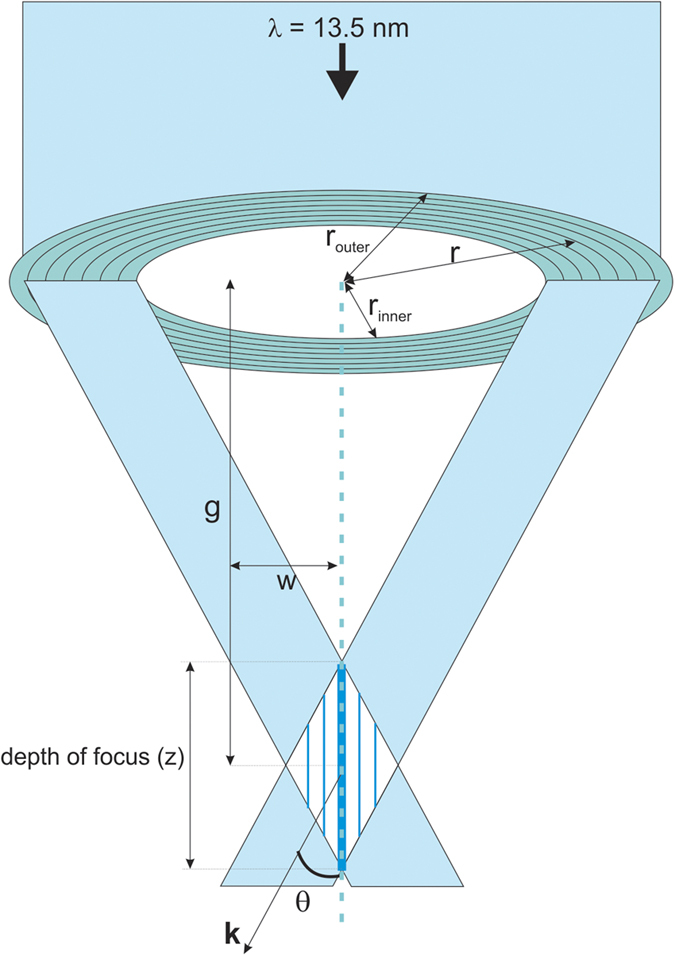
Schematic of Bessel beam formation showing the diffraction and interference of two beams from either end of the ring grating for simplicity. The interference pattern is a 2D Bessel function of the first kind, located at the working distance or gap *g*.

**Figure 2:**
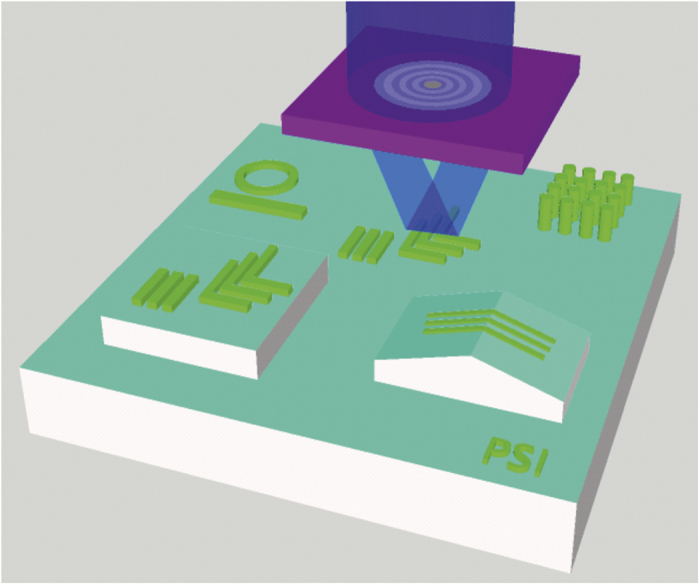
Bessel beam used as a serial writing method of arbitrary nanostructures over topographical surfaces.

**Table 1:** Bessel beam range for mask with 300 nm period calculated using equations 1 and 5.

	Min value (mm)	Optimal value (mm)	Max value (mm)
Gap for 1st order interference	1.45	2.00	2.56
Gap for 2nd order interference	0.72	1.00	1.27
